# Phosphate solubilizing *Aspergillus Niger* PH1 ameliorates growth and alleviates lead stress in maize through improved photosynthetic and antioxidant response

**DOI:** 10.1186/s12870-024-05361-5

**Published:** 2024-07-08

**Authors:** Iqbal Hussain, Muhammad Irshad, Anwar Hussain, Muhammad Qadir, Asif Mehmood, Muneebur Rahman, Abdulwahed Fahad Alrefaei, Mikhlid H. Almutairi, Sajid Ali, Muhammad Hamayun

**Affiliations:** 1 Department of Botany, Abdul Wali Khan University, Mardan, 23200 Pakistan; 2 Institute of Biological Sciences Sarhad University, Peshawar, Pakistan; 3 Department of Zoology, College of Science, King Saud University, Riyadh, Saudi Arabia; 4 Department of Horticulture and Life Science, Yeungnam University, Gyeongsan, Republic of Korea

**Keywords:** Heavy metals, Sequence homology, *Parthenium hysterophorus*, *Aspergillus niger*, Antioxidant system, Rhizospheric, Bio-fertilizer, Physio-hormonal

## Abstract

Among the several threats to humanity by anthropogenic activities, contamination of the environment by heavy metals is of great concern. Upon entry into the food chain, these metals cause serious hazards to plants and other organisms including humans. Use of microbes for bioremediation of the soil and stress mitigation in plants are among the preferred strategies to provide an efficient, cost-effective, eco-friendly solution of the problem. The current investigation is an attempt in this direction where fungal strain PH1 was isolated from the rhizosphere of *Parthenium hysterophorus* which was identified as *Aspergillus niger* by sequence homology of the ITS 1 and ITS 4 regions of the rRNA. The strain was tested for its effect on growth and biochemical parameters as reflection of its potential to mitigate Pb stress in *Zea mays* exposed to 100, 200 and 500 µg of Pb/g of soil. In the initial screening, it was revealed that the strain has the ability to tolerate lead stress, solubilize insoluble phosphate and produce plant growth promoting hormones (IAA and SA) and other metabolites like phenolics, flavonoids, sugar, protein and lipids. Under 500 µg of Pb/g of soil, *Z. mays* exhibited significant growth retardation with a reduction of 31% in root length, 30.5% in shoot length, 57.5% in fresh weight and 45.2% in dry weight as compared to control plants. Inoculation of *A. niger* to Pb treated plants not only restored root and shoot length, rather promoted it to a level significantly higher than the control plants. Association of the strain modulated the physio-hormonal attributes of maize plants that resulted in their better growth which indicated a state of low stress. Additionally, the strain boosted the antioxidant defence system of the maize there by causing a significant reduction in the ascorbic acid peroxidase (1.5%), catalase (19%) and 1,1-diphenyl-2 picrylhydrazyl (DPPH) radical scavenging activity (33.3%), indicating a lower stress condition as compared to their non-inoculated stressed plants. Based on current evidence, this strain can potentially be used as a biofertilizer for Pb-contaminated sites where it will improve overall plant health with the hope of achieving better biological and agricultural yields.

## Introduction

The modern world is facing many problems regarding pollution, among them heavy metals pollution is of great concern, that spread broadly around the world due to rapid urbanization and industrialization. The modern world from last few decades is going toward global climate change that lead to different kinds of pollution including air, water and soil [1]. Among heavy metals, Pb is enormously poisonous for plants and other organisms including humans [2].

Sources of Pb to soil and water include municipal sewage, paper and mash, Pb based paints, mining and refining activities, fuel and explosives [3]. Pb are mainly absorbed through roots and potentially poses several negative impacts on the physiochemical attributes of the host plants, lead to reduction in growth and finally death of the host plant [4]. Pb toxicity include suppression of enzymatic activities [5], impacting membrane permeability to nutrients and osmoregulation of the cell [6], reducing absorption potential of essential nutrition [7], deteriorating photosynthesis and transpiration and enhanced the oxidative stress through enhancing the synthesis of reactive oxygen species (ROS) [8], that adversely affect seed germination, development, growth, yield and other physiological activities of the host plant [9]. On the flipside, it also leads to carcinogenesis, gastro, respiratory, cardiovascular, neurodevelopmental, behavioural changes and urinary system ailments in humans [10].

To cope with heavy metal toxicity from the environment various types of chemicals, physical and biological mechanisms are used. Among them physicochemical mechanism are laborious, costly, time consuming and revealed mechanical complexity in the environment as compared to biological method [11].

For the removal of toxic organic and inorganic pollutants from soil different phytoremediation procedures are use globally. Phytoremediation is a cost effective, durable, viable and green technology with slow remediation of metal but effect by climate of particular area. In such scenario, use of microorganism especially rhizospheric microbes are used to mitigate HMs and other contaminated from the environment [12]. These microorganisms perform various defense mechanisms including production of metabolites, phytohormones, macronutrients solubilization, fixation of nitrogen. Furthermore, the rhizospheric microbes have the potential to improve immunity of symbiotic partner through the release of several phytotoxins, siderophores production, boosting antioxidant system by improve the biosynthesis of different antioxidants and biosynthesis of various group of low molecular weight organic compounds their release to environments and accumulation in various parts, helps the host plant to rescue their growth and alleviate various biotic and abiotic stresses [13].

Maize (*Zea mays* L.) is an essential edible flowering plant in the Poaceae family, widely cultivated during spring and summer. It ranks third among global cereal crops, serving as a crucial food source for humans and animals. *Z. mays* is rich in carbohydrates, minerals, protein, vitamin B and iron making it an excellent addition to the human diet [2].

The current research aims to assess the potential of the isolate PH1 to alleviate the Pb stress in *Z. mays*, modulation in physicochemical attributes of the host in metal and strain inoculated condition and the role of strain in enhancing the remediation potential of the host.

## Research methodology

### Isolation and preliminary screening of rhizospheric fungi

For isolation of rhizospheric fungi, *P. hysterophorus* plants were collected from Pb polluted sites of District Dir (Latitude 34^∘^ 45′ N to 35^∘^ 45′ N and Longitude 71^∘^ 30′E to 72^∘^ 30′ E) located in the northern hilly regions of *Khyber Pakhtunkhwa*, Pakistan. The selected plants were uprooted and packed quickly in sterilised plastic bags, transferred to Plant Microbes Interaction (PMI) laboratory in the Abdul Wali Khan University Mardan (AWKUM). The collected soil samples were processed within 24 h to overcome microbial contamination and not to lose our targeted fungal species.

For isolation of rhizospheric fungi serial dilution method by K Geetha, A Rajithasri and B Bhadraiah [14] was used. Plant roots almost 10 g were thoroughly shaken to remove the bulk soil followed by shaking of roots in sterilized distil water to make suspension. The soil suspension was diluted by serial dilution method and makes dilutions (10^− 1,^ 10^− 2^, 10^− 3^, 10^− 4^, 10^− 5^ and 10^− 6^). Furthermore, from each dilution aliquots of almost 100 µL were spread on Hagem medium by streaking method followed by incubation at 27 ℃ for seven days. Finally, the independent fungal strains that appeared on hagum plates were purified by subculturing method. Subculturing was repeated until the establishment of pure cultures which were preserved by preparing their slants on PDA medium [15] and by preparing its 50% glycerol stock.

### Screening the isolates for Pb tolerance

All the fungal isolates were sub cultured in Czapek medium by exposing to elevated concentration of Pb (0.1, 0.2, 0.4, 0.6, 0.8 and 1 g/L) to determine alleviation potential of the isolates [16]. Furthermore, inoculate almost 4 mm inoculum into 250 mL flask having 100 mL Czapek medium with and without Pb stress i.e., 0–1 g/L. All the inoculated flasks were transferred to shaker and incubate at 27 ℃ and 121 rpm for 10 days. After incubation, filtered the fungal biomass and filtrate through Whatman No 1 filter paper and monitored their fresh and dry weight. The strains that have the capability to grow in Czapek medium under said concentration of Pb were used for further experiments.

### Phosphate solubilization assay

For p-solubilizing assay, Pikovoskaya’s Agar (PVK) medium was used containing per litre 10 g dextrose, 0.5 g Ammonium sulphate (NH_4_)_2_SO_4_, 15 g Agar, 0.2 g Sodium chloride (NaCl), 0.1 g Magnesium Sulphate (MgSO_4_.7H_2_O), 0.2 g Potassium chloride (KCl), 0.5 g Yeast extract, 0.0001 g Manganese sulphate (MnSO_4_), 0.0001 g Iron sulphate (FeSO_4_.7H_2_O) as well as 5 g tricalcium phosphate Ca3(PO4)5, as insoluble phosphate source. All fungal isolates having potential of producing clear zone due to solubilisation of tricalcium phosphate (Ca_3_PO_4_) as insoluble phosphate source were selected as potent phosphate solubilizers [17].

Using the following formula of V Kumar and N Narula [18] to measure Phosphate solubilization index (SI) of selected fungal isolates up to six days.

$$\text{SI}\,=\,(\text{C}\text{o}\text{l}\text{o}\text{n}\text{y} + \text{H}\text{a}\text{l}\text{o} \text{z}\text{o}\text{n}\text{e} \,\text{d}\text{i}\text{a}\text{m}\text{e}\text{t}\text{e}\text{r})/\left(\text{C}\text{o}\text{l}\text{o}\text{n}\text{y} \,\text{d}\text{i}\text{a}\text{m}\text{e}\text{t}\text{e}\text{r}\right)$$

### Screening of fungal filtrate and plant materials for metabolites production

All the fungal isolates were sub cultured in Czapek medium followed by shaking incubation for 7 days, at 28 ℃ and 121 rpm. After incubation for seven days, the fungal biomass was filtered through Whatman No 1 filter paper. The fungal mycelium of selected isolates was used for plant growth promoting assay and culture filtrate (CF) of the isolates for stress related metabolites including phytohormones and different enzymes.

### Plant bioassay

#### Growth promotion and pb stress alleviation in *Z. mays* by PH1

Growth promotion and Pb stress alleviation of PH1 was checked on *Z. mays*, for that healthy and uniform size seeds Gulabati variety, purchased from local market were thoroughly surface sterilized. Moreover, sowing five seeds per treatment in decontaminated plastic pots containing per pot 200 g of sterilized soil (25% organic matter, 25% sand and 50% soil) in growth chamber (Model; LGC-5101 G). The fungal treatment was done by inoculating 1 g of fungal biomass in 100 g of autoclave soil (1: 100). The experiment was design in the following way.

(i) Control (*Z. mays* without fungal treatment and Pb stress).
(ii) Treatment 1 (Pb stress i.e.,100, 200 and 500 ug/g of soil).
(iii) Treatment 2 (*Z. mays* inoculated with PH1 only).
(iv) Treatment 3 (*Z. mays* inoculated with PH1, along with different concentration of lead stress as mentioned above).

When *Z. mays* seedlings got established under the condition i.e., 67% humidity, light intensity of 35,000 lx, 13 h photoperiod and temperature 28 ℃, the host plants were supplemented with 100, 200 and 500 ug/g of Pb stress. After 34 days of plant germination, seedlings were harvested and different growth parameter including root, shoot length, fresh and dry weight of all the treated and control plants were analysed. Similarly, the total chlorophyll and carotenoids content as well as biochemical analysis of all treated and untreated groups of plants were also recorded spectrophotometrically.

#### Determination of total flavonoids and IAA

The procedure of M El Far and H Taie [19], with minor modification was used to quantify flavonoids in plant materials and fungal CF. Further, fresh leaves (5 g) of *Z. mays* were grinded in 50 mL of 80% ethanol. While in the case of fungal CF, 1 mL of supernatant was mixed through the same mentioned procedure. To remove cellular debris the resultant extracts were centrifuged at 1000 rpm for 10 min. Approximately, 0.1 mL of 10% AlCl_3_, 0.1 mL of 10% potassium acetate, and 4.3 mL of 80% methanol were added to 1 mL of the extract. The resultant solutions were mixed well and kept for incubation at room temperature for colour development. Taking OD of resultant mixture at 415 nm against 80% methanol as a blank solution. Total flavonoids were determined as ug/mL for fungal CF and µg/g of plant extract.

For quantification of IAA in plant material and fungal CF, the colorimetric procedure of A Mehmood, A Hussain, M Irshad, M Hamayun, A Iqbal and N Khan [20] was used with minor modification. To prepare the samples for analysis, approximately 1 mL of sample was mixed with 2 mL of Salkowski reagent. The samples were kept for incubation at room temperature for at least 30 min for color development. Optical density was determined at 530 nm against blank having Salkowski reagent and expressed as µg/mL for fungal CF and ug/g of plant extract.

#### Determination of total phenolics contents

The technique EA Ainsworth and KM Gillespie [21] was used for the estimation of the total phenolic contents with slight modification. Approximately, 1 mL of the fungal CF was taken in a test tube and mixed with 10 mL of 70% ethanol. For extraction of phenols from leaf, crushed 1 g of leaf in 70% ethanol to make extract. Both the reaction mixture was thoroughly centrifuged at 5600 rpm for 10 min. Further, take 0.5 mL of supernatant from the mixture and carefully add 2 mL of 20% sodium carbonate and 0.5 mL of folin reagent. The reaction mixture was diluted through distilled water and reached final volume to 3 mL. The mixture was then incubated at dark condition for 2 h, the colour of the reaction mixture change to blue that illustrate the presence of phenols. Take OD at 650 nm against blank having folin as a reference solution. The quantity of phenols were show in µg/ml for fungal CF and µg/g of plant extract [22].

#### Determination of soluble sugar and proline contents in CF and plant materials

The procedure of M DuBois, KA Gilles, JK Hamilton, Pt Rebers and F Smith [23] with slight changes were adopted for determination of soluble sugar in CF of all the isolates and plant materials. The fungal CF of all the isolates and leaves extract (0.5 g of plants fresh leaves was grinded in 10 mL autoclaved dH_2_O) was centrifuge at 3000 rpm for 5 min. Thoroughly take 0.5 mL of sample and add 10 µL of 80% phenol, 1 mL absolute H_2_SO_4_ to the solution. The solution was carefully mixed and incubate at 25 ℃ for 10 min. OD was taken at 520 nm against blank having benedict as a reference solution.

Proline contents of samples (fresh leaves and fungal filtrate) using the protocol of LS Bates, Ra Waldren and I Teare [24]. Take almost 0.1 mL of fungal CF and homogenized with 4 mL of 3% sulfo-salicylic acid, while for plant sample 0.3 g of fresh leaf sample was ground in 4 mL of 3% sulfo-salicylic acid and centrifuged at 3000 rpm for 15 min. After centrifugation, add 2 mL of acid ninhydrin to 2 mL of the supernatant and incubate the mixture on water bath for 1 h at 98 °C followed by cooling. After proper cooling of reaction mixture add 4 mL of toluene for extraction of proline contents. OD was taken at 520 nm against blank having toluene.

#### Determination of SA in fungal CF and plant material

For determination of SA, take 100 µL of fungal CF and add mix with 2.9 mL Fecl_3_ (0.1%) solution. While from leaf 0.25 g was crushed in ethanol, followed by centrifugation at 10,000 rpm for 10 min. From ethanolic supernatant take 100 µL of the sample and add 0.1% Fecl_3_ (2.9 mL) and reached the final volume 3.0 mL. The appearance of violet colours show the formation of complex among SA and Fe^3+^ ions [25]. The OD was noted through spectrophotometer at 540 nm against blank having freshly prepare FeCl_3_.

#### Determination of total lipids and proteins

To determine total lipids, 0.2 mL of sample was taken from filtered fungal CF, while for plant samples, 0.2 g of leaves was ground in extraction buffer with 2 mL of 21% chloroform and 1 mL of methanol, followed by filtration of the mixture from Whatman No 1 filter paper to remove plant debris, and adding 1 mL of ethanol and 2 mL of chloroform. After thoroughly shaking the solution to dissolve it properly, add 0.8 mL (0.73%) of NaCl. The lowermost layer that contains lipid was taken and processed for lipids quantification by using phosphor-vanillin reagent [26]. The lowest layer was shifted to a 25 mL beaker and keep it on water bath for 5 h at 50 to evaporate. After that 5 mL of concentrated H_2_So_4_ was added and thoroughly mixed by shaking and kept in boiling water bath for ten minutes followed by cooling. After that to 2.4 mL of sample, add equal volume of phosphorus vanillin reagent. The OD of the solution was taken at 490 nm against blank having vanillin reagent as reference and quantified in unit of µg/g for plant materials and µg/mL for fungal CF.

The quantification of total proteins in the samples were performed according to the procedure of [27]. While for quantification of protein in plant samples the protocols of O Lowery, N Rosebrough, A Farr and R Randall [28], was used with slight modifications. OD of the reaction mixture was taken at 650 nm against blank having folin reagent. Various concentrations of Bovine Serum Albumin (BSA), 20, 40, 60, 80 and 100 µg/ml was used to make standard curve.

### Effect of pb toxicity on antioxidant system of the host

The free radical scavenging activity was recorded using DPPH [29]. Take 1 mL of fungal CF, while from leaves samples, 0.1 g was homogenized in 1 mL of methanol and add 1 mL of 0.004% of DPPH stock solution, gently homogenized the solution followed by incubation in dark for almost half hour. After 30 min incubation the colour of the reaction mixture changes and OD was recorded at 517 nm against blank having substrate.

$$\div\,DPPH=\left(1-\frac{AE}{AD}\right)\times 100$$

Further, AE illustrate the absorbance of DPPH solution having selected sample while AD shows the absorbance of DPPH stock solution only as a blank.

AAO activity was determine according to the procedure of K Asada [30]. For APX quantification, centrifuge the CF of selected strains at 3000 rpm for 7 min, while for plant 0.3 g of fresh leaves were homogenized in 3 mL of phosphate buffer. Take 200 µL of supernatant having enzyme and add 1.5 mL of 50 mM phosphate buffer, 1.5 mL of 0.5 mM ascorbic acid and lastly 1.5 mL of 0.1 mM of hydrogen peroxide. The OD of the reaction mixture was recorded at 290 nm against standard containing 1.5 mL of H_2_O_2_, ascorbic acid peroxidase and phosphate buffer each.

CAT Activity was quantified by the method of R Radhakrishnan, AL Khan, SM Kang and I-J Lee [31]. Thoroughly, 0.1 g of leaves was homogenized in 1 mL of phosphate buffer, followed by centrifugation at 11,000 rpm for 15 min at 4 ºC. Take 40 µL of sample from both fungal CF and plant samples and mixed with 400 µL of 15 mM H_2_O_2_. After thoroughly mixing the mixture, add 2.6 mL of sodium phosphate buffer and the OD was recorded at 240 nm against standard solution (having 400 µL of 15 mM H_2_O_2_ and 2.6 ml of phosphate buffer). Further, one enzymatic unit was assumed as the concentration of enzyme required to decrease the absorption by 0.05 unit at 240 nm.

### Genomic DNA extraction and fungal identification

The protocols of ST Khan, S Takaichi and S Harayama [32] was adopted for fungal DNA extraction and polymerase chain reaction (PCR). PH1, the most potent strain on the basis of Pb stress mitigation, production of metabolites, phosphate solubilization, and growth promotion, was identified by sequencing the internal transcribed region (ITS) of 18S rDNA. About 200 mg freeze dried mycelium of PH1 was ground in 500 µL of buffer (sodium-dodecyl-sulphate (SDS), 0.5 M Tris-HCl and 0.1 M NaCl at pH 8 and vortexed for 10 to15 seconds, heated for 30 minutes at 65°C, then centrifuge the mixture at 11000×g for 5 minutes. Equal volume of supernatants was gently mixed with an equal concentration of phenol, chloroform and isoamyl alcohol (25:24:1) followed by centrifugation at 10000 rpm for 5 minutes. The aqueous layer was mixed with chloroform-isoamyl alcohol (24:1) and centrifuged for 5 minutes at 10000 rpm, take the supernatant and thoroughly mixed with equal amount of concentrated ethanol incubated the mixture for 1 hour at 4°C and centrifuged at 14000 rpm for 10 minutes for DNA precipitation. The DNA was amplified with ITS (the internal transcribed region (ITS) of 18S rDNA, with universal primers ITS5 5’ (TCC GTA GGT GAA CCT GCG G) 3’ and ITS4 5’ (TCC TCC GCT TAT TGA TAT GC) 3’) regions via PCR. The reaction mixture consists of 20 µL (1 µL of each primer, 10µL PCR master mix, 1 µL of DNA sample, and 7 µL of dd H_2_O). The process of PCR consists of an initial denaturation for 2 min at 94 °C, primer annealing at 55 °C for 1 min and polymerization for 5 min at 72 °C. A total of 35 cycles were run. The PCR product first sequence and then subjected for homology search through using online tool NCBI BLAST (http://www.ncbi.nlm.nih.gov/BLAST/). Phylogenetic study was occur through MEGA-7 software [33].

### Determination of pb through atomic absorption spectrophotometer

For the estimation of Pb in fungal biomass of selected isolate and plant parts treated with said concentration of Pb, 0.5 g of oven dry biomass of plant and fungal isolate was subjected to acid digestion. For acid digestion add 4 mL of concentrated nitric acid (HNO_3_), to both of dry samples and boil the mixture for 30–45 min. After cooling of mixture, add 1 ml of 70% HClO_4_ and boiled gently till the appearance of dense white fumes. Again, the mixture was cool and diluted further by adding distil water to reached final volume up to 25 mL, followed by filtration from Whatman No 42 filter paper. The Pb concentration in both samples was quantified through an atomic absorption spectrophotometer (Perkin–Elmer model 700, MA, USA) following the protocol of [34].

### **Analysis of photosynthetic contents in*Z. mays***

The method of S Maclachlam and S Zalik [35], was used for total chlorophyll and carotenoids contents in the extract of fully expanded leaves before harvesting of plant. Approximately 0.3 g of fresh leaves were homogenised in 5 mL of (80%) acetone follow by centrifugation at 1000 rpm for 5 min. The supernatant of the mixture, washed 3 times using 1 mL of (80%) acetone and reached the final volume of reaction mixture up to 7 mL by acetone. The OD was taken at 640 nm, 646 nm, and 663 nm against 80% acetone as a standard solution.

### Statistical analysis

All the experiments were performed in three replicates. The data was analysed statistically through one-way analysis of variance (ANOVA) followed by Duncan’s multiple range test by using the statistical software (IBM SPSS Statics v.26.0) at *p* ≤ 0.05. The graph was plotted by using Graph pad prism (Version 9).

## Results

### Isolation and preliminary screening of rhizospheric fungi

**Fig. 1:**
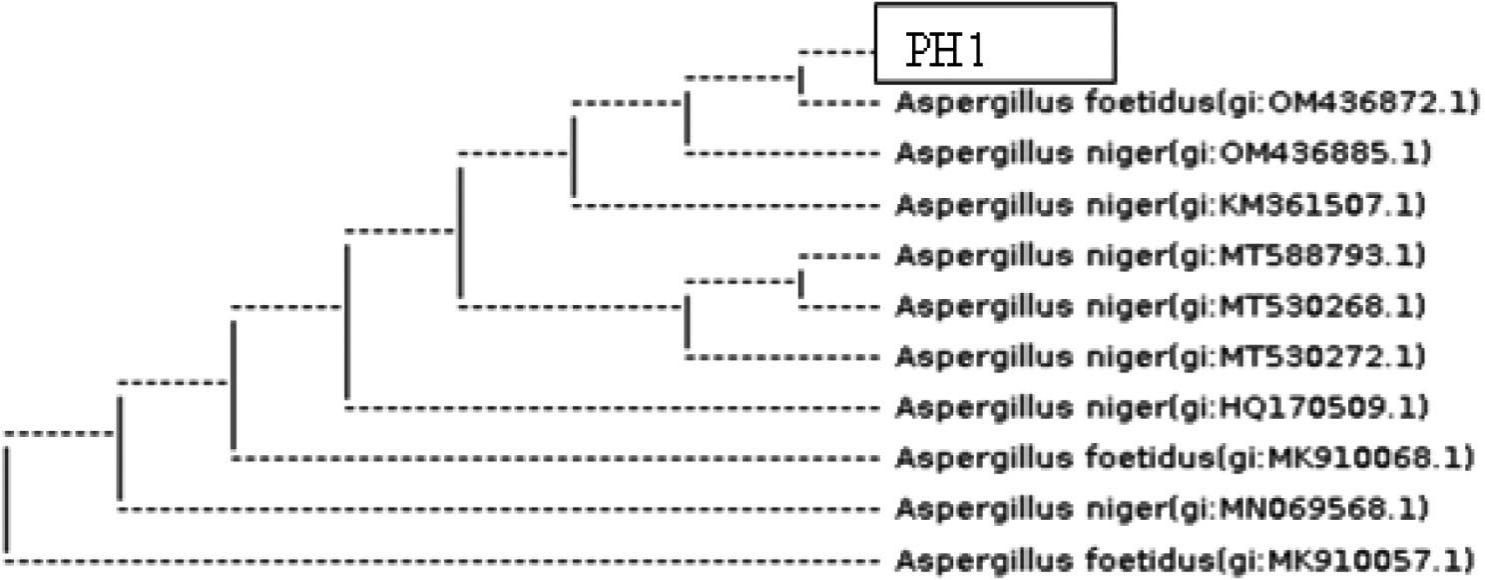
*A. niger* grown on PDA medium (**A**), PVK medium making the holozone (**B**), halo zone and colony diameter (**C**) and phosphate solubilization index (**D**), of *A. niger* grown in PVK medium for 7 days. Bars show mean of triplicates with standard error (SE). Means followed by different alphabets in lowercase represent difference from each other at *p* ≤ 0.05

A total of 11 morphologically different rhizospheric fungal strains were initially isolated from young and healthy plant of *P. hysterophorus* from Pb contaminated sites of Khall District (lower) Dir, Khyber Pakhtunkhwa Pakistan on hagum medium. All the fungal isolates were initially selected due to their morphological differences from one another and serially numbered i.e. from PH1 to PH11. Further, all the isolates were initially screened for plant growth promoting and Pb alleviating ability in Czapek medium as well as on *Z. mays* seedling. Among the total isolated strains PH1 inoculated on PDA medium (Fig. 1A), showed p-solubilization, boosting antioxidant system of the host and alleviate Pb stress up to 1 g/L. The selected fungal isolate produced growth promoting phytohormones and stress related metabolites in appreciable quantities.

### Screening of isolates for P-solubilization

To assess P-solubilization potential of isolated strains, inoculate 7 days old colony on Pvk medium. Phosphate solubilizing microbes have the potential of making a clear zone due to solubilization of bound TCP (Fig. 1B). Among the potent strains maximum colony and halazone diameter (4.2 and 2.3 cm) was noted for PH1 on day 6 of incubation (Fig. 1C). Furthermore, highest value (1.67 cm) of solubilization index (SI), was noted for PH1 on day 4 of inoculation (Fig. 1D). Interestingly the toxicity of Pb cannot affect the solubilization potential of selected fungal strain.

### Tolerance index of PH1 under Pb stress

**Fig. 2:**
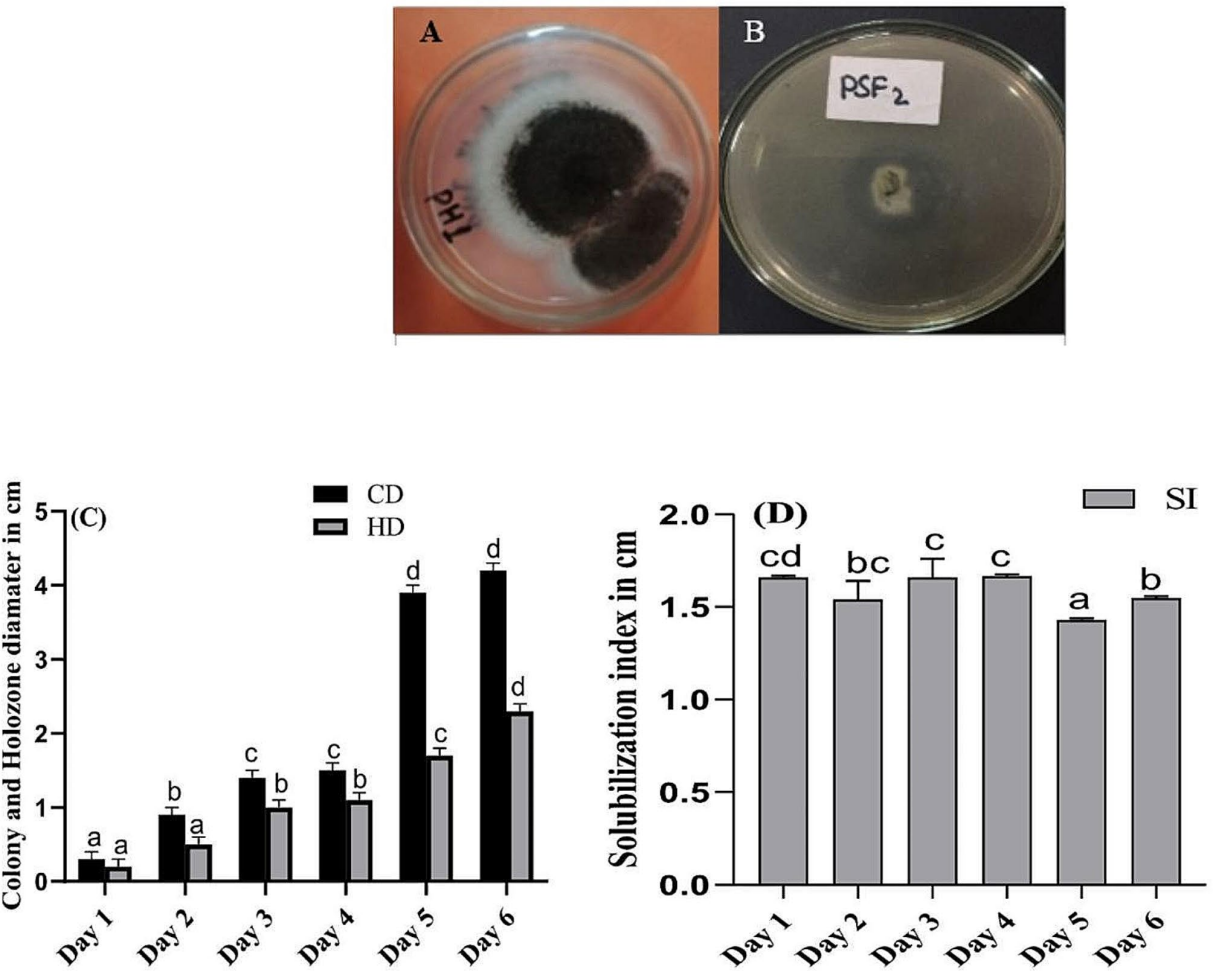
Tolerance index (**A**), fresh weight and dry weight (**B**), while accumulation of Pb in fungal biomass (**C**), of *A. niger* grown in Czapek medium at 27 ℃ and 121 rpm for 10 days. Bars illustrate means of triplicate with SE. Means followed by various letters are significantly difference from one another at *p* ≤ 0.05

In the current findings fresh and dry weight of PH1 was monitored. It has been observed that by increasing the concentration of Pb in Czapek medium (Fig. 2A), up to 0.5 g/L the strain could grow without any significant effect on fresh and dry weight, while further increase beyond the said concentration of Pb, sharp decline was recorded for both fresh and dry weight. However, for fresh weight as compare to control 9.4% while for dry weight 90.4% reduction was noted in highest concentration of Pb stress i.e., 1 g/L (Fig. 2B).

### Accumulation of Pb in fungal biomass

In the present study it was observe that, the amount of Pb per gram accumulated by PH1 increased with increasing the concentration of Pb in the medium (Fig. 2C). Moreover, the selected fungal strain on exposure to 1 g/L of stress, accumulate about 401 µg/g of Pb, which were the highest among the different treatment of Pb. Accumulation of Pb in fungal biomass was significantly increased with increasing concentration of Pb.

### Determination of metabolites in the CF

**Fig. 3:**
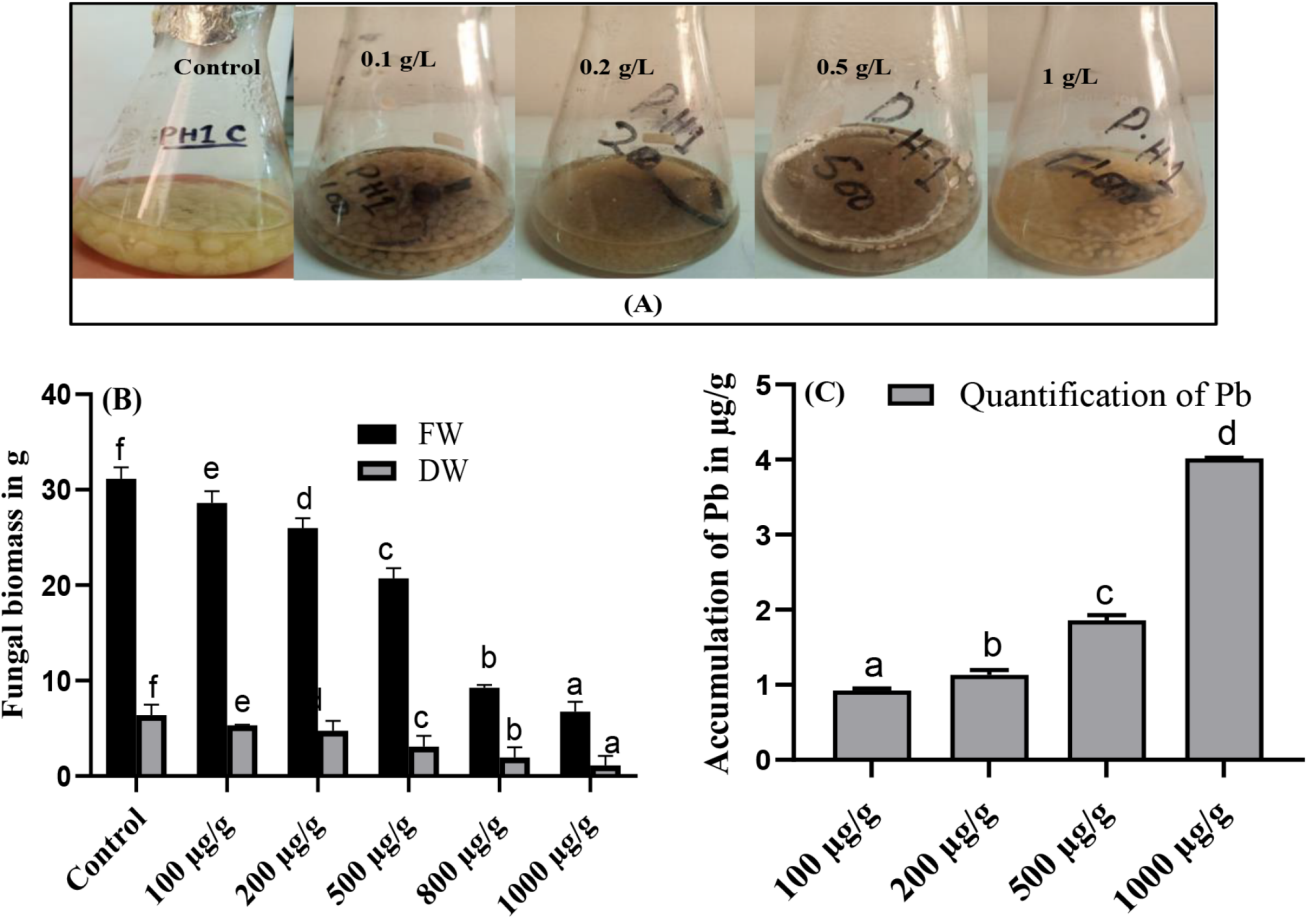
Effect of different concentrations of Pb on the production of flavonoids, lipids, sugar and Indole acetic acid (**A**), protein, salicylic acid and phenolics (**B**), of *A. niger* grown in Czapek medium at 27 ℃ and 121 rpm for 10 days. Bars illustrate means of triplicates with SE. Means followed by various alphabets in lower case represent significant difference from each other at *p* ≤ 0.05

In the present study overall, the production of flavonoids, lipids, sugar and IAA contents in the CF of PH1 were increased by exposing them to increasing concentration of Pb. Moreover, as compare to the CF of fungal control, 364, 1102, 364 and 368% increase was noted in the CF of *A. niger* on exposing to 1 g/L of Pb stress for flavonoids, lipids, sugar and IAA respectively (Fig. 3A). Furthermore, the concentration of protein, SA and phenol was increased significantly in the CF of PH1 by increasing concentration of Pb. Additionally, as compare to control, 433, 157 and 608% increase was noted for protein, SA and phenolics, in the CF of PH1 on exposing to highest toxicity of Pb (Fig. 3B).

### Identification of fungal isolate PH1

**Fig. 4:**
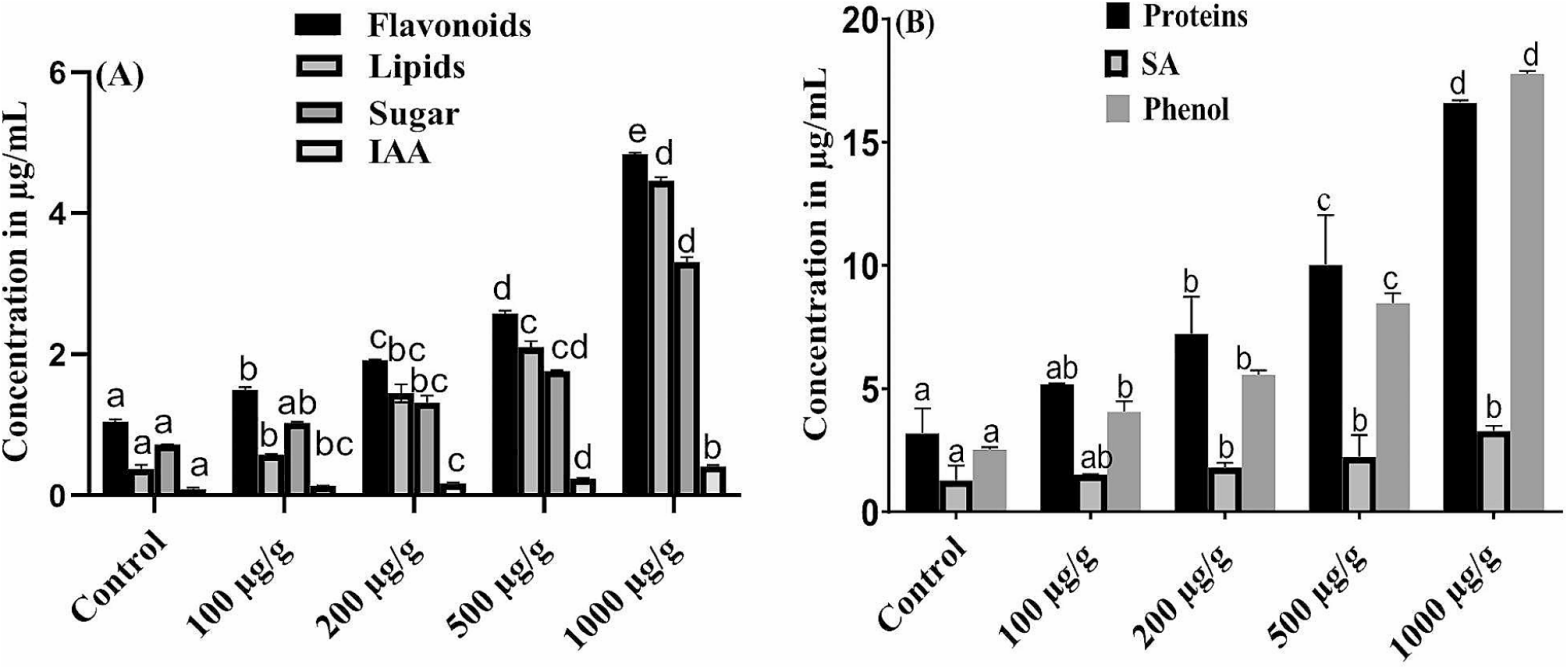
ITS based phylogenetic analysis of fungal isolate *A. niger* (PH1) by neighbor-joining (NJ) method. By using MEGA 7 software the evolutionary analyses were conducted

After removing the low-quality sequence with bioinformatic software (Finch TV), the forward and reverse strands were aligned with codon code aligner. The sequence was then blast in NCBI nucleotide blast. The strains (approximately 10 strains) with higher homology were selected and the sequence were downloaded. Further, the neighbour joining (NJ) method was used to assess phylogenetic analysis of selected fungal strain PH1 through Mega-X software. The phylogenetic tree was built from 11 taxa the ITS sequences was aligned with 100 bootstrap replications. The strain was selected through the BLAST search, illustrate the maximum sequence homology similarity. Results of BLAST search indicate maximum sequence similarity (100%) among the selected isolate PH1 and *A. niger* (Fig. 4), under accession number (PP621893) strain belong to family *Aspergillaceae*.

### Effect of Pb on antioxidant system of *A. Niger*

**Fig. 5:**
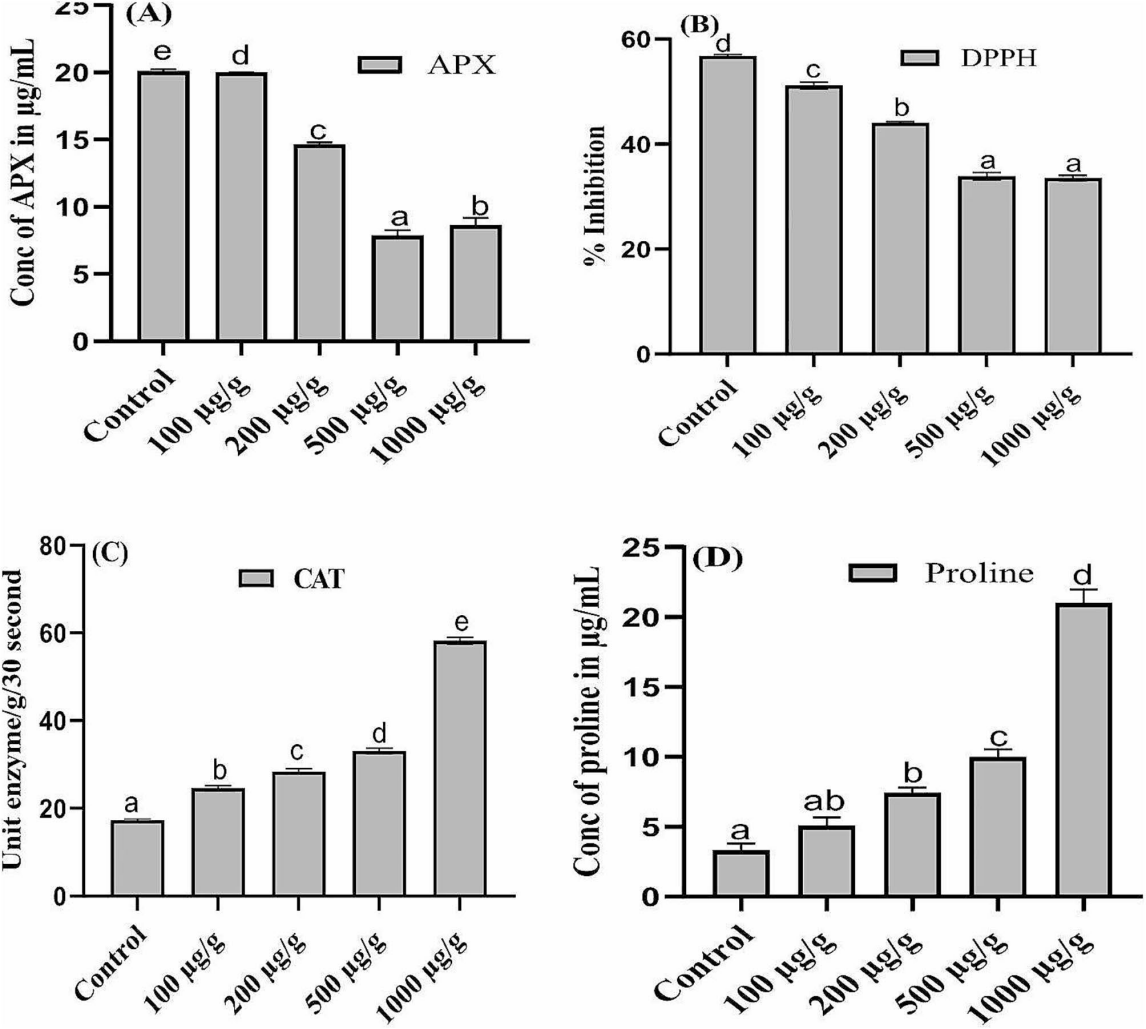
Effect of different concentration of Pb on the production of ascorbic acid peroxidase (**A**), 1,1-diphenyl-2 picrylhydrazyl radical scavenging activity (**B**), catalase activity (**C**), and concentration of proline (**D**), of *A. niger* grown in Czapek medium at 27 ℃ and 121 rpm for 10 days. Bars illustrate average of triplicates with SE. Means data followed by different alphabets are significantly difference from each other at *p* ≤ 0.05

The production of APX was significantly decrease by increasing the concentration of Pb as compared to the CF of *A. niger* under control condition. Moreover, lowest quantity with 65% decrease was recorded in the CF of *A. niger*, exposing to highest concentration of Pb stress as compared to the CF of *A. niger* under control condition (Fig. 5A).

DPPH assay was performed, and was noted that % inhibition was significantly decrease by increasing the toxicity of Pb along with *A. niger* (Fig. 5B). Highest % inhibition with 171% increase, was noted in the CF of *A. niger* on exposing to highest Pb stress as compare to the CF of *A. niger* under control condition.

Overall, it is noted that the concentration of CAT and proline in the CF of *A. niger* was increased by increasing the concentration of Pb. Furthermore, highest quantity of CAT and proline with 241 & 566% increase was noted in the CF of *A. niger* at 1 g/L of Pb stress compare to the CF of *A. niger* under control condition (Fig. 5C and D).

### Plant growth promoting and Pb alleviating potential of *A. Niger*

**Fig. 6:**
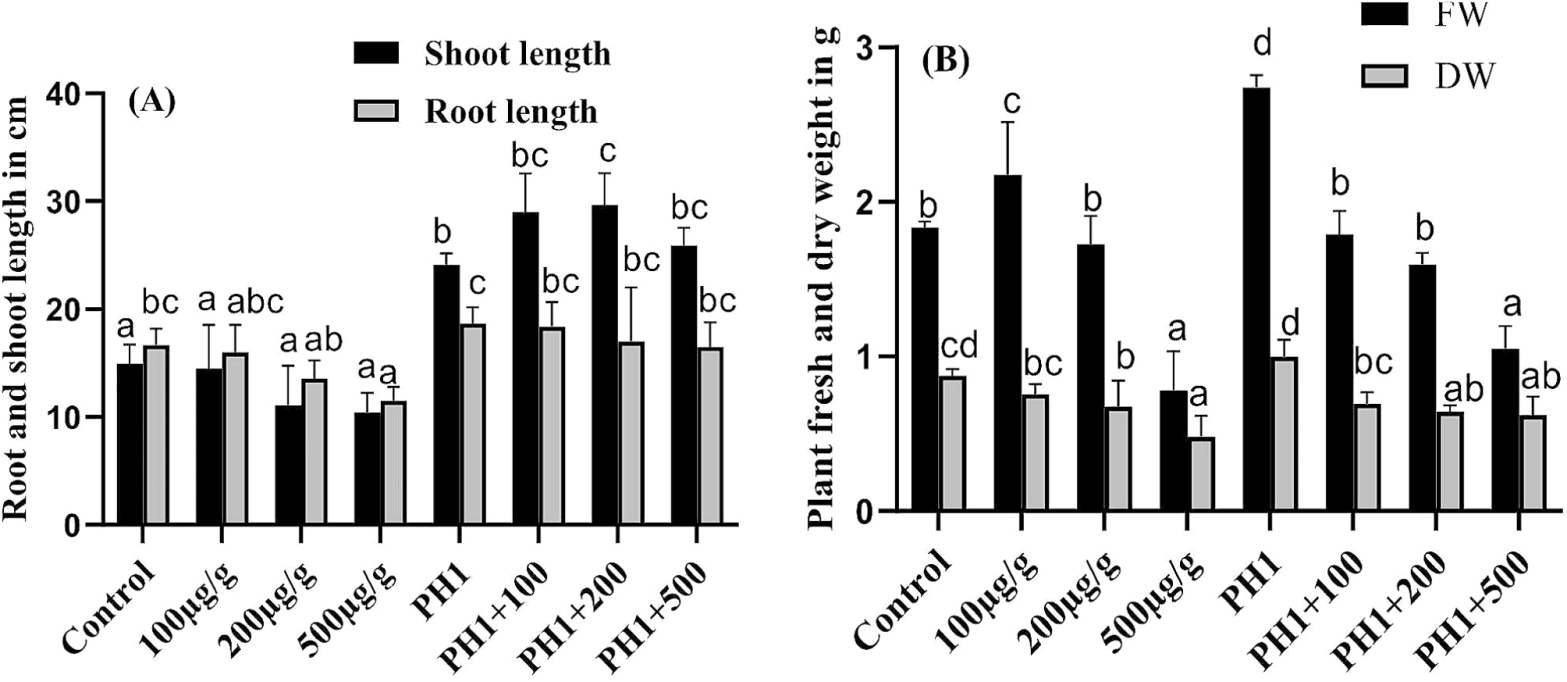
Effect of Pb stress of *Z. mays* plants inoculated with or without *A. niger* on shoot and root length (**A**), fresh weight and dry weight (**B**). The bars show the average data of triplicates with SE. Bars labelled with different alphabets show significant difference from each other at *p* ≤ 0

Exposing *Z. mays* plant to various concentration (100, 200 and 500 µg/g) of Pb stress a dose dependant decrease was noted in root shoot length and fresh and dry weight. On inoculation of *A. niger* to *Z. mays* plant along with Pb, alleviate the Pb toxicity by promoting all growth attributes. Further *Z. mays* plant, when inoculated with *A. niger*, promote the root (18 cm) and shoots (24 cm) as compared to seedlings under control conditions having root (16 cm) and shoots (14 cm) with 12.5 & 41.66% increase respectively (Fig. 6A). Similarly, the fresh and dry mass was noted in *A. niger* inoculated plant (2.7 & 0.9 g), which were higher as compare to un-inoculated control plant (1.8 & 0.8 g), with 50 & 12.5% increase for both fresh and dry mass of *Z. mays* (Fig. 6B). Moreover, as compare to control and Pb stress plants the growth parameter including root and shoot length as well as fresh and dry weight was clinched up significantly in those plants that are inoculated with *A. niger* and co-inoculated with different concentration of Pb.

### Determination of chlorophyll and carotenoids contents in *Z. mays*

**Fig. 7:**
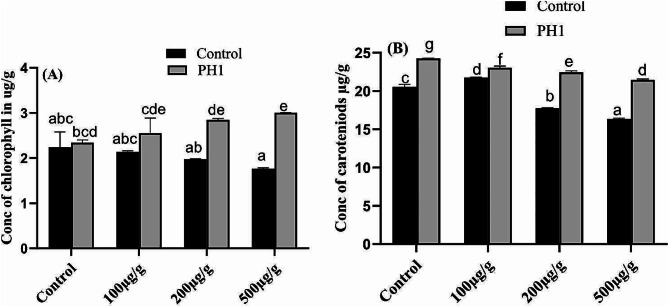
Effect of varying concentration of Pb and *A. niger* on production of chlorophyll (**A**) and carotenoids (**B**), of *Z. mays* plant. Bars represent means of triplicates with SE. Means data followed by different letters are significantly difference from one another at *p* ≤ 0.05

The effect of Pb and potential of *A. niger* to enhance chlorophyll and carotenoids content by mitigating metal toxicity are shown in (Fig. 7A and B). In comparison to control plant the chlorophyl and carotenoids contents of Pb treated plants were significantly decline. On the other hand, both chlorophyll and carotenoids contents were restored by inoculating the host plant with *A. niger* along with Pb. Moreover, as compare to control plant, the chlorophyll and carotenoids contents of *A. niger* treated plants were increased 102 & 97% respectively. Similarly, compare to 500 µg/g of Pb treated plants 168 & 114% increase was noted in the plants that are co-inoculated with *A. niger* having same Pb stress. Lowest chlorophyll and carotenoids were recorded in host plants treated with higher doses of the Pb i.e., 500 µg/g.

### Determination of IAA flavonoids and phenols contents in *Z. mays*

**Fig. 8:**
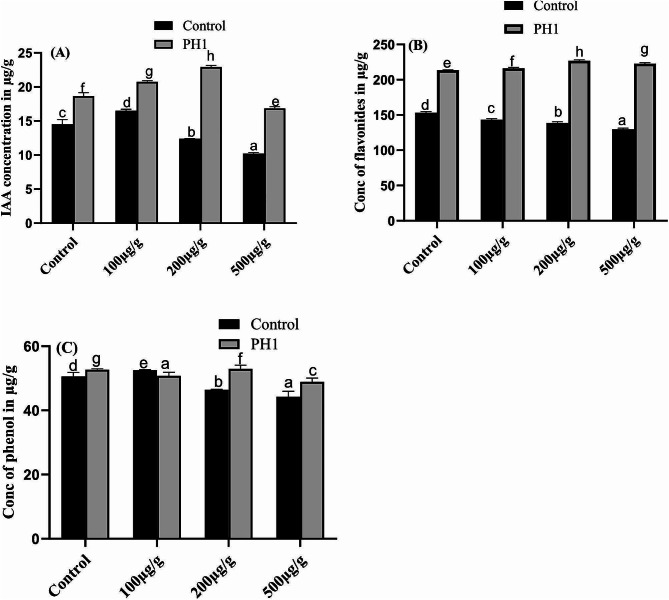
Effect of varying concentration of Pb and *A. niger* on production of IAA (**A**), flavonoids (**B**) and phenols (**C**) of *Z. mays* plants. Bars represent means of triplicates with SE. Means data followed by different letters are significantly difference from one another at *p* ≤ 0.05

Highest concentration of IAA and flavonoids were recorded in 200 µg/g of Pb along with *A. niger* treated *Z. mays* plants, that were further decrease sharply for IAA (Fig. 8A) and slightly for flavonoids (Fig. 8B) by increasing Pb toxicity. Furthermore, the IAA contents increase at 100 µg/g of Pb followed by a gradual decline whereas, the later trend was also followed by flavonoids. Overall, both IAA and flavonoids concentrations were increased significantly by inoculated the host plant with *A. niger* as well as co-inoculated with Pb.

Different kinds of phenolic compounds are synthesised by plants during stress condition to cope the toxicity of Pb. In present study compare to control seedlings the concentration of phenols production was increased with increasing the concentration of Pb except in 100 µg/g of Pb stress. On the other hand, inoculation of both *A. niger* and Pb to *Z. mays* seedlings, the production of phenols was first decrease in 100 µg/g of Pb stress compare to *A. niger* inoculating plants then increase slightly by increasing Pb stress and finally reduced significantly on exposing to highest Pb stress (Fig. 8C). Highest value 52.9 µg/g with 0.43% increase was recorded in seedlings exposed to 200 µg/g of Pb stress along with *A. niger* associated *Z. mays* plants.

### Determination of protein, lipids and sugar contents in *Z. mays*

**Fig. 9:**
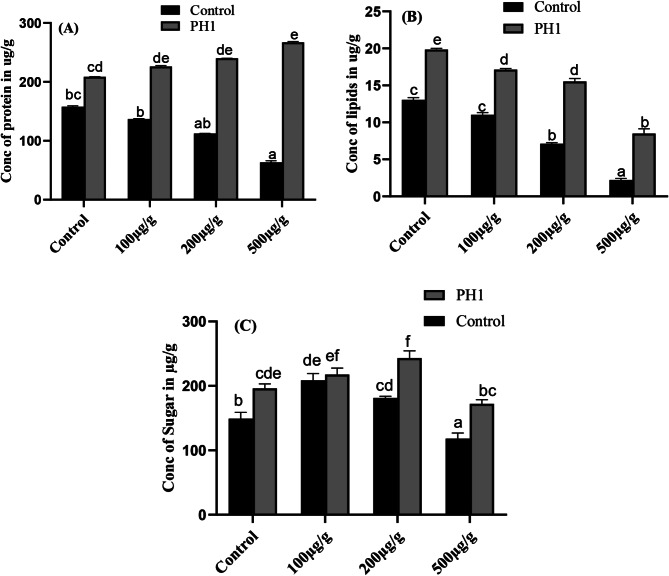
Effect of varying concentration of Pb and *A. niger* on production of protein (**A**), lipids (**B**) and sugar (**C**) of *Z. mays* plants. Bars represent means of triplicates with SE. Means data followed by different letters are significantly difference from one another at *p* ≤ 0.05

In present study it was observed that the concentration of protein in *Z. mays* leaves decreased significantly with increasing the concentration of Pb as compare to control seedlings. On the other hand, when the host plants were inoculated with *A. niger* as a bio-inoculant the total protein content increase and further increase by increasing the concentration of Pb. Highest protein contents 267 µg/g with 28% increase was recorded in 500 µg/g of Pb along with *A. niger* as compare to plants that are inoculated with *A. niger* (Fig. 9A).

In present research findings, significant decline was recorded in the concentration of lipids for both Pb treated plants and plants that are treated with *A. niger* along with different concentration of Pb as compare to control and *A. niger* treated plants. Moreover, highest concentration of lipids 19 µg/g with 52% increase was recorded for plants that are inoculated with selected fungal strain compare to control plant. while lowest quantity 2.2 µg/g of lipids with 83% decrease was recorded in plants that was exposed to 500 µg/g of Pb stress as compared to control seedlings (Fig. 9B).

*A. niger* improved soluble sugar in *Z. mays* seedlings under different concentration of Pb compare to plants under control condition. In present study, it was noted that total soluble sugar increases in 100 µg/g of Pb stress compare to control plant, while declined by further increasing the concentration of Pb. Similarly, as compare to control plants the concentration of soluble sugar increased in *Z. mays* seedlings that were treated with *A. niger* along with 100 & 200 µg/g of Pb stress and declined by further increasing Pb stress (Fig. 9C). Moreover, highest quantity (243 µg/g) of sugar with 23.9% increase was noted in plants that were treated with *A. niger* along with 200 µg/g of Pb stress as compared to seedlings treated with *A. niger*. While lowest quantity (118 µg/g) of sugar with 20% decrease was noted in 500 µg/g of Pb treated plant as compared to *Z. mays* under normal conditions.

### Quantification of catalase in *Z. mays* under pb stress

**Fig. 10:**
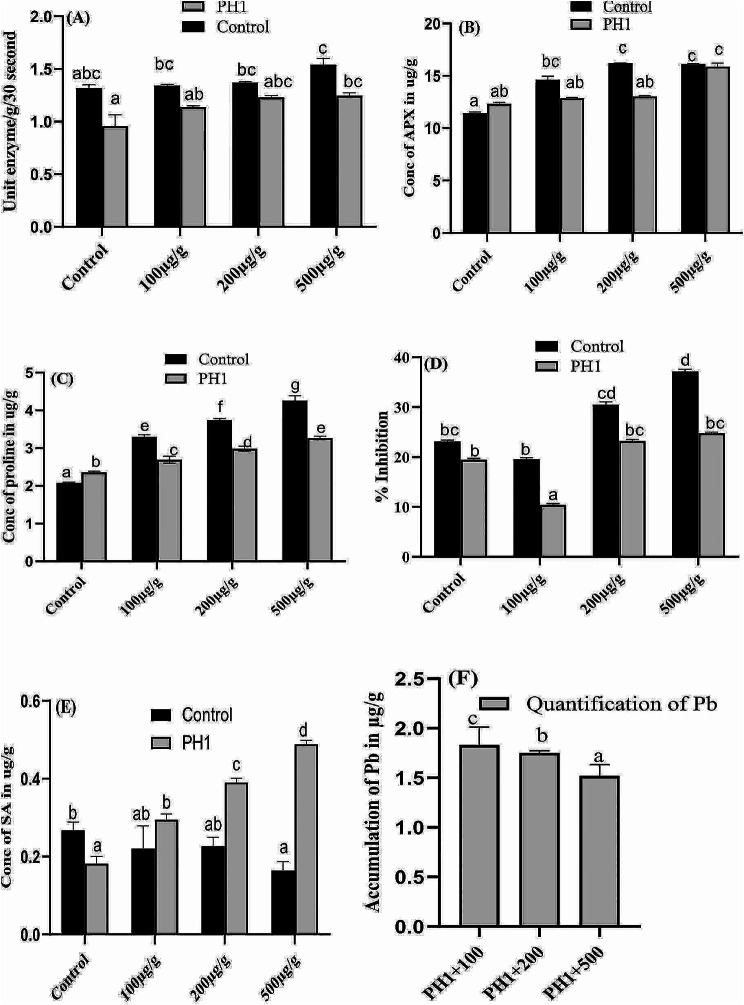
Effect of varying concentration of Pb and *A. niger* on production of catalase (**A**), ascorbic acid peroxidase (**B**), proline (**C**), DPPH radical scavenging activity (**D**), SA (**E**) and accumulation of Pb in plants are shown in (**F**). Bars illustrate average data of triplicates with SE. Means data labelled with various alphabets are significantly difference from each other at *p* ≤ 0.05

In present research work, highest amount of catalase (1.54 µg/g, a 17.5% increase) was recorded in seedlings exposed to 500 µg/g of Pb stress, compare to control seedlings, while lowest quantity (0.95 µg/g with 27.4% decrease) was noted in the plants treated with *A. niger* compared to control plants (Fig. 10A). Moreover, it is noted that the concentration of catalase increased significantly with increasing Pb stress, both alone as well as in combination with *A. niger*, compared to both fungal-treated and non-treated control plants.

### Determination of APX and proline contents in *Z. mays*

Total ascorbic acid peroxidase production was significantly higher in Pb-inoculated plants compared to non-inoculated control plants. However, metal toxicity did not negatively affect APX activity as shown in Fig. 10B. Moreover, it was noted that, compared to both fungal and plant controls, the APX content was increased by increasing the concentration of Pb alone or along with *A. niger*. The highest quantity of peroxidase (15.8 µg/g, a 28.6% increase) was noted in plants treated with 500 µg/g of Pb along with *A. niger*, compared to fungal-treated control plant.

The concentration of proline increases significantly as the dose rates of Pb increases. Interestingly, an increase of 58.9, 80.6 and 105% was recorded in proline content in *Z. mays* leaves at 100–500 µg/g of Pb stress, compared to control plant (Fig. 10C). On the other hand, when *A. niger* was inoculated as bio-inoculant, a significant increase was recorded in proline content as compared to control plants. Overall, compared to control plant and plants inoculated with *A. niger*, the concentration of proline increases with increasing Pb concentrations, as well as in those plants treated with both fungi and Pb.

### Quantification of % DPPH in *Z. mays* under pb stress

The DPPH radical scavenging assay was measured by exposing the host plant to different concentration of Pb. In present investigation the radical scavenging potential of the *A. niger* was decrease by exposing the plants to 100 µg/g of Pb stress and then increase significantly by increasing the concentration of Pb. Maximum scavenging activity (37 µg/g) with 60% increase was noted in 500 µg/g of Pb stress compare to seedlings under control condition. Moreover, as compare to both plant and fungal control, the % DPPH activity was first decrease in seedlings exposed to 100 µg/g of Pb alone or in combination with *A. niger*, then increase continuously by increasing the concentration of Pb (Fig. 10D).

### *A. Niger* improved SA in *Z. mays* plants under pb stress

In present findings maximum amount 0.48 µg/g of SA with 168% increase was recorded in 500 µg/g of Pb stress along with *A. niger* compare to plants inoculate with *A. niger.* On the other hand, lowest quantity 0.16 µg/g with 38% decrease was noted in highest metal concentration as compare to control seedlings (Fig. 10E). Furthermore, it was noted that SA contents of the leaves are reducing significantly by increasing the concentration of Pb while increasing significantly by co-inoculation with *A. niger.*

### Accumulation of Pb in *Z. mays* associated with *A. Niger*

The quantity of Pb accumulated by various parts of the *Z. mays* plant decreases with increases the concentration of Pb in the medium (Fig. 10F). For instance, the seedling exposed to 100 µg/g of Pb stress accumulated about 1.83 µg/g of Pb in their biomass, which was the highest among the seedlings exposed to different concentrations of Pb. Further, accumulation of Pb in various plant parts was declined significantly in seedlings treated with increasing concentration of Pb along with *A. niger*.

## Discussion

Heavy metal pollution is a threat to modern humanity either in one way or the other. Pakistan, as a under developed country, faces the issue with impact of multi-folds due to the improper agricultural practice and unavailability of techniques and measures to remove the contaminants from the environment. These toxic HMs from various sources, combine with different water bodies, reached to soil and lead to reduction of plant growth and development as well as initiates serious health problems in humans consuming the product of such contaminated soil [13]. A novel strategy of bioremediation is extensively in practice to reclaim the contaminated site taking the advantage of physiological processes carried out by these microbes. In the last two decades, several other measure including phytoremediation and chemical remediation are also in practice [36].

In the current scenario, a total of eleven rhizospheric fungi were isolated from the rhizosphere of *P. hysterophorus*. Among the total isolates *A. niger* strain PH1 was able to solubilized the inorganic phosphate, effectively manage to alleviate Pb stress, and modulate host physiology. The solubilization of certain nutrient and making them biologically available for plants help them to mitigate the biotic stress by scaring some important nutrient for the pathogens. R Mendes, P Garbeva and JM Raaijmakers [37] reported that, phosphate solubilizing microbes formed clear zone around their colony by solubilizing bound phosphate through production of different acids that lower the pH of the medium and enhance plant growth due to availability of essential nutrients and also help eradicate the pathogens.

Similarly, the tolerance index 1 g/L was noted for *A. niger* with 401 µg/g of Pb accumulation in the fungal biomass. A Mishra and A Malik [38] suggesting that, fungi can neutralize the toxicity of heavy metals due to surface precipitation, immobilization through oxalate production, bioaccumulation, and making complex compounds with organic acids including metal oxides, metal hydroxide, moolooites. Growth and morphology of *A. niger* were also affected by elevated Pb concentration which is believed to be due to inhibition of enzymatic activities by interaction with protein, displace for important metals ions, cause distraction of biological membrane, cause oxidative stress through production of hydroxyl or superoxide radicals and reducing bioavailability of nutrients [39].

Initial screening of the CF of *A. niger* for metabolites production shows that the selected fungal strain has potential to produce various metabolites in different concentration on exposing to various concentration of Pb. Among them, the production of IAA in fungal CF was significantly increased with increasing Pb toxicity in liquid culture medium. This is because the selected fungal strain has the potential to adopt Pb stress as compared to other strains, having less production or inhibition of their growth. Higher IAA production not only help the host to growth efficiently but also has been reported to have a stress mitigating role by initiating root growth and enhancing the compartmentalization of the toxic metals. According to A Mehmood, N Khan, M Irshad, M Hamayun, I Husna, A Javed and A Hussain [40] endophytic fungi have the capability to produce significant quantity of IAA during stress condition. Similarly, *A. niger* enhanced the production of flavonoids and phenolics under elevated Pb stress, that enhancing fitness of *A. niger* to endure Pb stress. The flavonoids and phenolics are stress related metabolites and also direct quencher of reactive oxygen species that are produced as a result of metal stress helping the host to grow normally. Similarly, to improve adapting strategies of *A. niger*, strain PH1 to mitigate Pb stress increased concentrations of phytohormones, secondary metabolites and antioxidant potential by producing CAT, APX, proline and % DPPH indicated that this rhizospheric fungal isolate could be valuable to mitigate Pb stress in plants. The modulation of antioxidant defence system help the host to mitigate the oxidative stress that prevent the lipid peroxidation of the biological membrane and maintaining the vitality of the cell. O Sytar, P Kumari, S Yadav, M Brestic and A Rastogi [41] suggested that, phytohormones regulate growth during HMs stress conditions through strengthen antioxidant system and metabolism as well as increase cell division, rate of transpiration, metabolism and assimilation of nitrogen, positively control ascorbate–glutathione pathway are some of vital factors for plant growth promotion. In fact, *A. niger* on exposure to elevated concentration of Pb produce different metabolites and stress related enzymes to strengthen the antioxidant system are important tool for host plant to cope the toxicity of Pb stress. While maintaining cell integrity, the host cell uses the reactive oxygen species as a signal molecule to activate the antioxidant defence system, which in first instance start deposition of lignin in higher quantities that act as first line of defence. On the other hand, the antioxidant system help to lower the secondary oxidative toxicity by lowering membrane lipid peroxidation which help to keep the essential ions in the cell and prevent their loss helping in maintaining the viability of the cell. BJ Lugtenberg, N Malfanova, F Kamilova and G Berg [42] propose that different plant associated microbes have the capability to absorb metabolites secreted by the fungal partner in the closed vicinity of the roots. Furthermore, with the intake of microbial secreted phytohormones, binding of SA Calcium dependent protein Kinases (CDPKs), triggering stress responsive downstream genes, Osmotins, Heat shock proteins (HSPs), that are used to contest a series of abiotic and biotic stressors.

The effect of *A. niger* was also checked in symbiosis with *Z. mays* for growth promotion and stress mitigation by adding above mentioned concentration of Pb. Higher levels of phytohormones and growth-related metabolites production help the host to accumulate higher fresh and dry biomass. Furthermore, all the growth parameter including root, shoot length and fresh and dry weight was decrease with increasing Pb toxicity. However, the adverse effect was decline by treating the host plant with *A. niger*. Moreover, as compare to control 12.5% and 41.6% increase was recorded in seedlings exposed to highest Pb stress co-cultivated with *A. niger*. Likewise, significant increase was noted in seedling fresh weight 2.7 g with 50% increase and dry weight 0.9 g with 12.5% increase as compare to control plants. This increase in the biomass is attributed to the symbiotic association of the fungal strains with the host plant. Similar results was reported with M Ikram, N Ali, G Jan, FG Jan, IU Rahman, A Iqbal and M Hamayun [43] and by observing that the growth parameter under salt stress were increased by inoculating the host plant with endophytic fungi. Similarly, M Kajla, VK Yadav, J Khokhar, S Singh, R Chhokar, RP Meena and R Sharma [44] reported that plant biomass significantly increases by treatment of fungal culture filtrate to host plant. Moreover, as compared to control and Pb treated plants significant increase was noted in chlorophyll and carotenoids contents of those plants treated with *A. niger* as well as co-inoculated with different concentrations of Pb. These results are similar to the finding of S Ullah and A Bano [45], by observing increase in chlorophyll content in the leaves of *Z. mays* following fungi inoculation even in the presence of different metal. The accumulation of ROS is more effectively scavenged through the enhanced accumulation of antioxidant enzymes including APX, DPPH and CAT that boosted the defence mechanism of host plant under stress condition. The elevated activities of antioxidant enzymes are the key sign for recognition of oxidative stress in plant M Anjum, R Miandad, M Waqas, F Gehany and M Barakat [46]. So, in present study, the *A. niger* helped the host plant to produce huge amount of antioxidant enzymes including CAT, APX, and % DPPH under Pb stress. The increase production of these enzymes scavenges the ROS, that assist the host plant to grow normally under different concentration of Pb stress. Besides, the production of antioxidant enzymes, the *A. niger* also produce proline, SA and sugar in their CF and also triggers the host for their production, proline contents during stress condition increase due to enhanced enzymatic activities because proline act as an antioxidant or osmolyte [2]. Moreover, microbes induce the production of SA in the medium on exposure to stress condition, that are reabsorb from culture and convert to SA based metabolites including siderophores [47]. The elevated level of SA based siderophores are used to produced iron and mitigate HMs by convert them to various less or non-toxic chemicals [48]. Similarly, elevated concentration of sugar are also a common indication of stress condition, [49]. In present study the concentration of IAA, significantly increase with increasing metal toxicity as compare to control plant. Plants produce various phytohormones in significant quantity to overcome the toxicity of stress. The role of phytohormones particularly IAA, is vital for attracting microbes from close vicinity of the root to establish beneficial symbiotic association and facilitate defensive response of the host through expression of several genes including IAA 30 [50]. Moreover, during stress condition plants also needs flavonoids and phenolics to cope metal toxicity by scavenging the toxic radical produced by metal toxicity [51]. In our case both the concentration of phenolics and flavonoids was increased with increasing the concentration of Pb. The current findings show correlation with the findings of FC Moreira-Vilar, R de Cássia Siqueira-Soares, A Finger-Teixeira, DM de Oliveira, AP Ferro, GJ da Rocha, LF Maria de Lourdes, WD dos Santos and O Ferrarese-Filho [52], who presented that during salt stress flavonoids in tomato plant increased significantly. High levels of phenolics, flavonoids, improve cell wall toughness by the formation of physical barrier that safeguard cells from HMs toxicity [53]. The strains also help the host plant to accumulate higher quantities of metal while growing normally. This was possible by the modulation of the phytohormones production including growth hormones like IAA, GA and stress related hormones like SA which help the host to grow normally in stress [54]. Along with that the modulation in the metabolites production also help the host withstand the higher accumulation of Pb while growing normally. Some of the metabolites acts as ROS quenchers while other acts as osmolytes preventing the primary metal and secondary oxidative stress while maintaining osmoregulation and homeostatic conditions [55–57]. Higher accumulation of the metal was attributed to higher compartmentalization as under higher hormonal condition higher cell division and growth occurs which help the host plant to distribute the metal and lessen their negative toxic effects [58, 59].

## Conclusion

The current studies have revealed the *A. niger* ability to alleviate lead toxicity, solubilize phosphorous and promote growth of *Zea mays* plant. Moreover, the interaction of selected fungal strain with host plant can significantly increase their nutritive quality and quantity. From agronomic perspective, the current research study is very important and will help to design a bio-fertilizer and bio-remediant in the form of *A. niger*. On environmental and economic perspective, *A. niger* are vital due to its low cost, sustainable way to manage the pollution and hence, environmentally friendly. From the current evidences it is concluded that the use of *A. niger*, is call of the day for the reclamation of heavy metal contaminated soil while acting as biofertilizer. Limitation to the study include the commercialization of the production using it as an effective biofertilizer and potent bioremediatory agent. Future perspective includes digging some effective ways to commercialize effective microbial consortia to effectively replace the synthetic fertilizer and remediate the environment in sustainable and natural way.

## Data Availability

The datasets generated and/or analysed during the current study are submitted in the GenBank repository under accession number PP621893. (https://www.ncbi.nlm.nih.gov/nuccore/PP621893).

## Abbreviations

Pb: Lead
PH1: Parthenium hysterophorus
APX: Ascorbic acid peroxidase
CAT: Catalase
DPPH: 1,1-diphenyl-2 picrylhydrazyl
ROS: Reactive oxygen species
HMs: Heavy metals
PMI: Plant Microbes Interaction
AWKUM: Abdul Wali Khan University Mardan
PVK: Pikovoskaya’s Agar
PDA: Potato dextrose agar
TCA: Tricalcium phosphate
SI: Solubilization index
CF: Culture filtrate
IAA: Indole acetic acid
OD: Optical density
SA: Salicylic acid
PCR: Polymerase chain reaction
ITS: Internal transcribed region
ANOVA: One-way analysis of variance
CD: Colony diameter
HD: Halazone diameter
NJ: Neighbour joining
SE: Standard error
